# The yeast dynamin-like GTPase Vps1 mediates Atg9 transport to the phagophore assembly site in *Saccharomyces cerevisiae*

**DOI:** 10.1080/27694127.2023.2247309

**Published:** 2023-08-17

**Authors:** Yan Hu, Fulvio Reggiori

**Affiliations:** a Department of Biomedicine, Aarhus University, Ole Worms Allé 4, 8000 Aarhus C, Denmark; b Aarhus Institute of Advanced Studies (AIAS), Aarhus University, Høegh-Guldbergs Gade 6B, 8000 Aarhus C, Denmark

**Keywords:** Autophagosome, autophagy, DNM2, phagophore, *Saccharomyces cerevisiae*, traffic

## Abstract

Macroautophagy/autophagy is a degradative pathway that plays an important role in maintaining cellular homeostasis in eukaryotes. During autophagy, cisternal compartments called phagophores are generated to sequester intracellular components; these structures mature into autophagosomes, which deliver the cargo into lysosomes/vacuoles for degradation. Numerous autophagy-related (Atg) proteins are part of the core machinery that mediates autophagosome biogenesis. Atg9, a lipid scramblase and the only multispanning transmembrane protein among the core Atg machinery, traffics between cytoplasmic reservoirs and the phagophore assembly site (PAS) to provide membranes, recruit other Atg proteins and rearrange lipids on the phagophore membrane. However, the factors mediating Atg9 trafficking remain to be fully understood. In our recent study, we found that the yeast dynamin-like GTPase Vps1 (vacuolar protein sorting 1) is involved in autophagy and is important for Atg9 transport to the PAS. Moreover, we showed that Vps1 function in autophagy requires its GTPase and oligomerization activities. Interestingly, specific mutations in DNM2 (dynamin 2), one of the human homologs of Vps1 that have been linked with specific human diseases such as microcytic anemia and Charcot-Marie-Tooth, also impairs Atg9 transport to the PAS, suggesting that a defect in autophagy may underlay the pathophysiology of these severe human pathologies.

Autophagy is a catabolic process conserved among eukaryotes. Cisternal structures called phagophores are formed *de novo* to either selectively or nonselectively sequester unwanted cellular material and deliver it, after maturation into autophagosomes, into the mammalian lysosome or the yeast/plant vacuole for degradation. The autophagy-related (Atg) proteins are essential for this pathway, which starts with the nucleation and expansion of the phagophore. Around 20 Atg proteins are considered the core Atg machinery, and they are subdivided into six clusters based on their functions. Atg9 is the only transmembrane protein among the core Atg machinery and belongs to the Atg9-containing vesicle cluster. The Atg9-containing vesicles play a seed role in both the nucleation of the phagophore and the assembly of the Atg machinery. Moreover, the expansion of the phagophore requires the scramblase activity of Atg9 to rearrange the lipids transferred from the endoplasmic reticulum by Atg2. In yeast, Atg9 is present in tubulovesicular compartments, also called the Atg9 reservoirs, which are derived from the Golgi apparatus and are recruited to the phagophore assembly site (PAS) upon the induction of the formation of an autophagosome (Figure 1). Atg9 is then recycled and reused after autophagosome completion (Figure 1). Although playing a central role in regulating the progression of autophagy, Atg9 trafficking remains to be fully understood.

Dynamins are GTPases that can remodel membranes and mediate fission, and they play an important role in trafficking in both the endomembrane system and organelle dynamics. In our study we explored whether the two yeast *Saccharomyces cerevisiae* dynamins, Vps1 (vacuolar protein sorting 1) and Dnm1 (dynamin-related 1), are involved in autophagy [1]. We found that Vps1 is not only involved in bulk autophagy, but also cytoplasm-to-vacuole targeting (Cvt) pathway, a biosynthetic selective type of autophagy that delivers hydrolases like the precursor form of Ape1 (aminopeptidase I) to the vacuole [1]. Autophagy can be subdivided into 6 steps: initiation, phagophore nucleation, phagophore expansion, phagophore closure into an autophagosome, autophagosome-vacuole fusion, and degradation and recycling of the autophagosomal cargo. Using electron microscopy and protease protection analyses, but also by localizing several GFP-tagged proteins, we found that Vps1 is involved in the early steps of autophagosome formation. This protein, however, localizes to the Atg9 reservoirs rather than the PAS. Indeed, Venus-based bimolecular fluorescence complementation and ascorbate peroxidase 2 (APEX2)-based proximity-dependent biotin labeling showed that Vps1 directly or indirectly interacts with Atg9 at the Atg9 reservoirs. Because Atg9 redistribution to the PAS is one of the events taking place when the Atg machinery assembles during the early steps of autophagy, we examined Atg9 trafficking by fluorescence microscopy using the standard yeast procedure known as the transport of Atg9 after knocking out *ATG1* (TAKA) assay. This analysis revealed that Vps1 is involved in Atg9 transport to the PAS and, by using specific point mutants, we also showed that the GTPase and oligomerization activities of Vps1, which are essential for its membrane remodeling, are required for this function in autophagy. Mutations in human dynamins are the cause of some neurological disorders and other pathologies. We found that insertion of several of the corresponding mutations in Vps1 blocked Atg9 trafficking and autophagy, suggesting that a defect in autophagy may be a contributing etiological factor in these diseases.

In summary, we show that the dynamin-like GTPase Vps1 associates with Atg9 at the Atg9 reservoirs to mediate Atg9 transport to the PAS [1] (Figure 1). This finding provides novel insights into the mechanism of autophagy, and is in line with studies performed in mammalian cells in which DNM2 (dynamin 2), one of the three mammalian dynamin isoforms that has previously been shown to be involved in autophagy, is required for ATG9A trafficking. However, there are still several questions regarding how Vps1 contributes to Atg9 trafficking. For example, it is unclear which membrane remodeling event is required for the Atg9 delivery to the PAS. The most plausible hypothesis is that Vps1 is required for the formation of the Atg9-containing vesicles from the Atg9 reservoirs or eventually the Golgi, or even the generation of the Atg9 reservoirs from the Golgi (Figure 1). However, it cannot be excluded that the Atg9-containing vesicles/Atg9 reservoirs must undergo a maturation event to make them primed to contribute to autophagosome biogenesis. Other scenarios are possible as well. Another open question is whether Vps1 directly binds Atg9 and whether this interaction is mediated by one or more other proteins that participate in Atg9 transport to the PAS, like Atg23 and Atg27, which form a complex with Atg9 and are involved in the biogenesis of the Atg9-containing vesicles. Thus, additional investigations are required to understand the function of Vps1 in autophagy and to have a better understanding of Atg9 trafficking. Interestingly, one of the Vps1 point mutants that we examined and that impairs Vps1 self-assembly and oligomerization, Vps1^I422G^, blocks autophagy without affecting Atg9 trafficking. This observation implies that Vps1 is probably involved in one or more additional steps of autophagy as well.

## Figures and Tables

**Figure 1:**
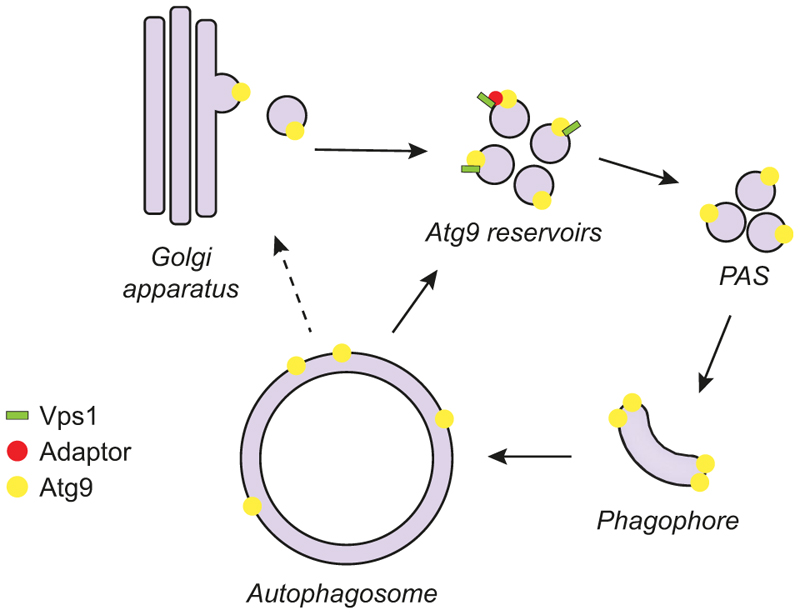
Possible role of the yeast dynamin-like GTPase Vps1 in Atg9 trafficking during autophagy. Vps1 directly or indirectly, via one or more adaptors, interacts with Atg9 at Atg9 reservoirs, which are derived from the Golgi apparatus. Vps1 mediates Atg9 transport from the Atg9 reservoirs to the PAS, although a role in sorting from the Golgi cannot be excluded. At the PAS, Atg9 provides the seed membrane to form the phagophore, but it is also playing a key role in recruiting part of the Atg machinery and rearranges lipids on the phagophore membrane to assist in expansion. Once the autophagosome is completed, Atg9 is recycled from the autophagosomal membrane or the vacuole upon fusion of the autophagosome with this organelle. It is unclear if this retrograde transport brings Atg9 back to the Atg9 reservoirs or to the Golgi, from where it returns to the reservoirs.

## Abbreviations

Ape1: aminopeptidase I
APEX2: ascorbate peroxidase 2
Atg: autophagy-related
Cvt: cytoplasm-to-vacuole targeting
Dnm1: dynamin-related 1
DNM2: dynamin 2
PAS: phagophore assembly site
TAKA: transport of Atg9 after knocking out *ATG1*
Vps1: vacuolar protein sorting 1

## References

[1] Arlt H, Raman B, Filali-Mouncef Y (2023). The dynamin Vps1 mediates Atg9 transport to the sites of autophagosome formation. J Biol Chem.

